# Human cytomegalovirus infection among treatment-naive HIV-1 infected patients in Ethiopia

**DOI:** 10.1371/journal.pone.0247264

**Published:** 2021-02-18

**Authors:** Mulugeta Kiros, Alene Geteneh, Henok Andualem, Derbie Alemu, Abebech Tesfaye, Dessalegne Abeje Tefera, Adane Mihret, Dawit Hailu Alemayehu, Andargachew Mulu

**Affiliations:** 1 Department of Medical Laboratory Science, College of Medicine and Health Sciences, Debre Tabor University, Debre Tabor, Ethiopia; 2 Department of Medical Laboratory Sciences, College of Health Sciences, Woldia University, Woldia, Ethiopia; 3 Department of Medical Laboratory Sciences, Arba Minch College of Health Sciences, Arba Minch, Ethiopia; 4 Armauer Hansen Research Institute, Addis Ababa, Ethiopia; Cleveland Clinic, UNITED STATES

## Abstract

Subclinical human cytomegalovirus (HCMV) replication is associated with immune dysfunction in immuno-suppressed antiretroviral therapy (ART) naive HIV infected individuals. No data is documented in Ethiopia so far concerning HCMV co-infection among HIV infected individuals. Hence, this study was aimed at generating data regarding the prevalence of active HCMV infection among treatment-naive HIV-infected individuals from Ethiopia. For this purpose, we enrolled 97 treatment-naive HIV infected study subjects in Addis Ababa from June to December 2018. ELISA and conventional PCR were performed consecutively to detect HCMV specific IgM antibody and HCMV DNA respectively. Of the 97 study subjects, 12 (12.4%) were positive for anti-CMV IgM antibodies but were not confirmed by PCR. With regard to the PCR positivity, 4/97 (4.1%) samples were positive for HCMV DNA. No statically significant associations were found between the dependent and independent variables. The presence of HCMV DNA in the current study highlights the need for a routine laboratory diagnosis for preventing HCMV disease among HIV-infected individuals early. Besides, the use of anti-CMV therapy for these CMV viremic individuals is also recommended as this can reduce the burden of CMV complications and consecutively prolonging the life of HIV infected individuals.

## Introduction

Opportunistic infections (OIs) remain a major concern in people living with Human Immunodeficiency Virus (PLWH) [1]. OIs in these individuals can be caused by viruses, bacteria, fungi, and protozoa [2]. Human cytomegalovirus (HCMV) is one of the highly prevalent human herpesviruses that cause OIs in PLWH. Although it has a global endemicity, it is more widespread in developing countries than in developed countries [2, 3].

In most cases, primary HCMV infection is asymptomatic in immunocompetent individuals, and the virus usually establishes a latent infection upon resolution of the acute phase [4]. However, in PLWH, this latent infection becomes reactivated and causes a systemic disease, which in turn causes high morbidity and mortality [4–6]. The effect is worse among HIV patients who are not on ART than those who are on ART [7]. In PLWH, CMV causes various life-threatening infections like retinitis, pneumonia, encephalitis, enteritis, etc. [1, 6]. These infections last a lifetime and can antagonize the immune system endlessly [8]. During infection with HCMV, specific antibodies against the virus are produced. The first type of antibody to develop is HCMV specific IgM, while IgG antibody is produced later. The CMV IgM produced then remains detectable for a longer period even after the resolution of a primary infection. Besides primary infection, IgM/IgG antibodies can also be detected during secondary infection either as re-activation or as re-infection [2].

Unless the virus is diagnosed early and appropriate treatment is given, HCMV viremia hastens the HIV disease progression by accelerating the immune system aging and eventually leads to death [9–11]. The two commonly used laboratory methods for the detection of HCMV among PLWH are PCR (that detects the virus replication) and serum immunological methods like ELISA (that detects antibodies produced against the virus) [1]. However, due to delayed CMV antibody production in PLWH that can lead to a false-negative result, PCR is the preferred diagnostic method for the detection of HCMV infection [1]. Since a late diagnosis of CMV infection is reported to cause dismal outcomes among PLWH, early diagnosis of the virus in these individuals is very critical for the management and monitoring of the infection [12].

Despite its cruciality, diagnostic testing for HCMV is not routinely done globally in PLWH [13]. In developing countries, the diagnosis and treatment of HCMV infection remain a challenge that has received little attention although the ART is scaled-up [14, 15]. Similarly, no study was done concerning HCMV infection among treatment-naive HIV infected people from Ethiopia as far as our knowledge is concerned. Therefore, this study was aimed at determining the prevalence of HCMV among treatment-naive HIV infected study subjects in Addis Ababa.

## Methods

### Study design and population

A cross-sectional study was implemented among 97 consented treatment-naive HIV-infected subjects in Addis Ababa from June to December 2018. Ethical approval was obtained from the Armauer Hansen Research Institute (AHRI)/ All Africa Leprosy Rehabilitation and Training Center (ALERT) ethical review committee (Protocol Number: PO16/18). All confirmed HIV-infected adult individuals (aged ≥18 years old) who attended the voluntary counseling test centers in Addis Ababa during the study period and willing to participate were consecutively recruited in this study.

### Sample collection and preparation

Blood (10 mL) was drawn from each patient into EDTA-treated tubes by trained medical personnel. Two hours after collection, the blood was then centrifuged for 10 minutes at the speed of 1200g (3000 rpm) following the WHO recommendation [16]. The plasma was aliquotted, transported to AHRI laboratory, and stored at -80°C until required for laboratory investigation.

### HIV-1 viral load measurement and HCMV serology

HIV-1 Viral load was first measured using the Abbott Real-time HIV-1 M2000rt (Abbott Laboratories, Abbott Park, USA). Plasma samples were then tested for the presence or absence of IgM antibodies using the commercially available anti-HCMV Enzyme-Linked Immunosorbent Assay (ELISA; Euroimmun, Germany) kit. The tests were performed and interpreted according to the manufacturer’s instructions.

### PCR amplification

DNA was first extracted from the participant’s plasma using QIAamp DNA Mini Kit (Qiagen, Hilden, Germany) following the manufacturer’s instruction. Plasma was used instead of peripheral mononuclear cells as this can avoid detection of latent HCMV genomes. Conventional PCR was then carried out in a 25 μL reaction mix consisting of 12.5 μl Platinum™ Hot Start PCR Master Mix (Invitrogen; the United States), 1μL of each HCMV forward primer (5’-TCGACGTTTCCACACAGACATG-3’) and HCMV Reverse primer (5’-GTGGTAGAAGCGGCGAAAGG-3’) that binds to the UL97 gene of the virus, 5.5μL molecular grade water, and 5 μL of template DNA. The thermal cycling condition includes initial denaturation at 95°C for 15 min, 35 cycles with denaturation at 95°C for 30 seconds, annealing at 60°C for 45 sec, and elongation at 72°C followed by a final extension at 72°C for 10 minutes [17]. Gel electrophoresis was run on 2.0% agarose gel for visualizing the amplified PCR products (729 bp) [17].

### Quality control

Socio-demographic and clinical data were collected using a pre-designed structured questionnaire. The data were then checked and entered in double to minimize error in data entry. The DNA extraction, master mix preparation, and PCR were performed in separate laboratory spaces to ensure the quality of test results. In each laboratory work, both positive control (NATCMV-0005; Helvetica Health Care, Geneva Switzerland) and negative control (molecular grade water) were run in parallel to make sure that there are no false positive and false negative PCR test results. Similarly, for the ELISA test, negative and positive controls that came with the kit were run parallel to the sample to make sure the reliability of the result. Overall, SOPs were strictly followed to ensure the consistency and precision of the test outcome.

### Statistical analyses

Data entry was performed using Epi data v3.1 software and statistical analysis was made using SPSS 25.0 software (SPSS Inc. the United States). A logistic regression model was used to check the associations between dependent and independent variables. A P value of <0.05 was considered statistically significant.

## Results

### Sociodemographic and clinical characteristics of study participants

Of the 97 consented HIV-1 infected study participants, about 59% (n = 57) of them were females, comprising a 1:1.43 male to female ratio (Table 1). Among the HCMV seropositive subjects, 58.3% (n = 7) of them were female while 41.7% (n = 4) were male. The ages of the study participants ranged from 19 to 57 years old. The average age of study participants was 32 years. Looking at the HIV-1 viral load level, more than half (50.8%) of the patients studied had a viral load of ≤ 100, 000 copies/ml (Table 1). Concerning HCMV IgM seropositive subjects, the majority (66%) of them had HIV-1 viral load > 100, 000 copies/ml (Table 1). Similarly, 50% of the HCMV DNA positive subjects had HIV-1 viral load > 100, 000 copies/ml (Table 1).

**Table 1:** Sociodemographic and clinical characteristics of study participants.

Characteristics		Frequency (N)	Percentage (%)	Individuals with HCMV ELISA positive (N)		Individuals with HCMV DNA (N)	
				(N)	(%)	(N)	(%)
**Sex**	Male	40	41.2	5	41.7	1	25
	Female	57	58.8	7	58.3	3	75
**Age Category**	18–28	38	39.2	5	41.7	2	50
	29–38	38	39.2	4	33.3	1	25
	39–48	15	15.5	1	8.3	0	0
	>49	6	6.2	2	16.7	1	25
**Current HIV-1 Viral load (Copies/ml)**	<2000	11	11.3	1	8.3	0	0
	2000–10000	9	9.3	0	0	0	0
	10001–100000	35	36.1	3	25	2	50
	>100000	42	43.3	8	66.7	2	50
**Occupation**	Unemployed	43	44.3	5	41.7	0	0
	Employed	54	55.7	7	58.3	4	100
**Marital status**	Married	43	44.3	3	25	1	25
	Single	28	28.9	4	33.3	2	50
	Stable relationship but not married	1	1	1	8.3	0	0
	Divorced	15	15.5	3	25	0	0
	Widowed/widower	10	10.3	1	8.3	1	25
**Educational status**	No schooling	17	17.5	2	16.7	0	0
	Primary	26	26.8	1	8.3	2	50
	Secondary	37	38.1	4	33.3	1	25
	College (diploma)	7	7.2	2	16.7	0	0
	University degree	10	10.3	3	25	1	25

### HCMV serology

Of 97 study participants, 12/97 (12.4%) of them showed HCMV IgM antibody positive results not confirmed by PCR test (Table 2). Although not statistically significant, the seroprevalence of the HCMV IgM antibody was slightly higher in the female participants (58.3%) than in males (41.7%). In addition, 66% of the HCMV IgM seropositive subjects had HIV-1 viral load > 100,000 copies/ml but the association was not statistically significant.

**Table 2:** Comparison of conventional PCR and ELISA for HCMV detection in plasma samples of the ART-naive HIV-1 infected individuals.

		Conventional PCR		
		Positive	Negative	Total
**IgM ELISA**	Positive	0	12	12
	Negative	4	81	85
	Total	4	93	97

### HCMV polymerase chain reaction

Considering the importance of CMV DNAemia as an indicator of active HCMV infection and marker of rapid HIV progression, we tested all the patient samples for the presence of HCMV DNA. HCMV DNA was detected in four (4/97, 4.1%) of the study participants. In terms of the sex distribution of the PCR positive samples, 75% (3/4) of them were female (Table 1). HIV viral copy of these positive samples was also to be between 17,800 and 182,040 copies/ml. Those PCR positive samples had negative serology results and vice versa (Table 2).

## Discussion

The early detection and intervention of opportunistic viruses such as HCMV among PLWH are very crucial for delaying HIV disease progression [9]. Albeit the high HIV prevalence, there are only limited studies done concerning HCMV infection in Africa. Similarly, there is no documented report regarding HCMV co-infection among treatment-naive HIV infected individuals from Ethiopia. Here we reported the HCMV IgM seroprevalence and HCMV viremia among treatment-naive HIV-infected individuals from Ethiopia using both ELISA and conventional. Since the demographics of the cohorts in this study is similar to the demographics of treatment-naive PLWH in Ethiopia, we believe that this study is reflective of the general population.

In line with studies from Nigeria (11.7) [2] and the USA (17%) [18], the HCMV IgM seroprevalence in the current study was 12.4%. However, the magnitude of the HCMV in the current study is higher than those that are reported from other countries; Serbia (7.1%) [19], Sudan (6.1%) [20]. This could be due to the difference in the study populations. Besides, the HCMV IgM seropositivity in the current study was not confirmed by PCR similar to other studies [19–21]. The inability to detect HCMV DNA in patients who were IgM positive might be due to the persistence of IgM antibodies for an extended period after primary infection [22, 23]. After the resolution of primary infection, HCMV specific IgM can be detectable for an average of 6 to 9 months [2].

Concerning the HCMV PCR in the current study, 4.1% (4/97) of the study subjects were HCMV PCR positive. This is consistent with the studies from South Africa, which were reported to be 5.2% [24], and India (7%) [25]. However, the current finding is lower relative to the findings from Tanzania (22.6%) [14], Kenya (17%) [26], and Cambodia 55.2% [27]. With respect to sex distribution, 75% of participants with confirmed HCMV DNA were females and this is in line with a previous study from Sudan [28].

In this study, the HCMV PCR positive samples were negative for ELISA IgM antibodies similar to other studies from Sudan [20, 29, 30]. The absence of HCMV IgM antibody in those PCR positive individuals could be due to the time lag between primary infection and IgM antibody production since IgM antibodies may remain undetectable because of delayed seroconversion [22, 23]. Besides, those PCR positive subjects might have reactivated HCMV infection, which usually does not induce a strong IgM response leading to a negative ELISA IgM result.

The presence of HCMV DNA (HCMV viremia), which is a better indicator of active HCMV infection [6], has been described as a well-known risk factor for mortality of treatment-naive HIV-infected individuals [24, 31]. Besides, the detection of HCMV DNA in patients with HIV has been serving as an AIDS-defining marker in the past. A study from Norway described that HCMV disease has served as the AIDS-defining diagnosis in 13 patients (6.1%) who died from AIDS [31]. Based on this, HCMV DNA detection among PLWH in the current study could be an early predictor of disease progression. A study from California also supports the hypothesis that HIV infected patients with DNAemia/positive plasma HCMV DNA were shown to be correlated with a 2.5-fold increased risk of death over 650 days [32]. Similarly, a study done in South Africa indicated that subclinical CMV viremia was an independent risk factor for mortality among adult males living with HIV in South Africa [33]. These findings suggest the need for early diagnosis and subsequent treatment of HCMV infection.

The prophylactic HCMV therapy has been shown to reduce the risk of CMV disease if initiated early upon HCMV infection diagnosis [34]. So far, there are four antiviral drugs licensed for the treatment of HCMV infections: ganciclovir, valganciclovir, foscarnet, cidofovir, and letermovir [35]. Due to the cost barrier, only ganciclovir was in use in developing countries in the past. However, valganciclovir is now started to be introduced in low and middle-income countries as it is available at a reduced price within the WHO essential medicines list [36, 37].

In this study, we have reported the HCMV seroprevalence and HCMV viremia among treatment-naive patients in Ethiopia for the first time. Apart from this strength, however, due to reagent shortages, we did not include a CD4 count that made a description of HCMV among PLWH in terms of immunosuppression difficult.

## Conclusions

The prevalence of HCMV infection according to the PCR test was 4.1%. As it was reported in the literature about the association of HCMV viremia and the development of CMV disease, a routine laboratory diagnosis is highly critical in preventing this disease ahead of time. Besides, the use of anti-CMV therapy parallel to ART for CMV viremic individuals is also recommended as this can reduce the burden of CMV complications and consecutively prolonging the life of PLWH. Moreover, we recommend further studies with a large sample size to be done at a national level to see the overall picture of HCMV-HIV co-infection at a national level, which in turn helps to develop national disease prevention and treatment plan and/or guideline.

## Abbreviations

HIV: Human Immunodeficiency Virus
ART: Antiretroviral Therapy
HCMV: cytomegalovirus
PCR: polymerase chain reaction
IgM: Immunoglobulin M
ELISA: enzyme-linked immunosorbent assay
PLWH: People Living With HIV
